# Trait-based modeling of buffalograss seed yield using UAV-derived plant height and canopy nitrogen concentration

**DOI:** 10.3389/fpls.2026.1802837

**Published:** 2026-05-05

**Authors:** Chu Wang, Xinyue Qu, Yuting Wang, Wouter H. Maes, Maona Li, Yan Sun

**Affiliations:** 1 College of Grassland Science and Technology, China Agricultural University, Beijing, China; 2 Cangzhou Academy of Agriculture and Forestry Sciences, Cangzhou, China; 3 Unpiloted Aerial Vehicle (UAV) Research Centre, Department of Plants and Crops, Ghent University, Ghent, Belgium

**Keywords:** buffalograss, nitrogen management, seed yield prediction, trait-based modeling, UAV remote sensing

## Abstract

Accurate seed-yield prediction is essential for optimizing nitrogen (N) management in buffalograss seed production. However, current UAV-based approaches often rely directly on vegetation indices (VIs), which provide limited physiological insight and not transfer well across growing seasons. To address this limitation, we developed a trait-based yield prediction methold that integrates UAV-derived plant height (PH) and canopy nitrogen concentration (CNC), representing crop structural and physiological status, respectively. Field experiments were conducted from 2022 to 2024 under seven N application rates. Using data from 2022 and 2023, we calibrated a quadratic PH-CNC model and then evaluated its predictive performance with an independent 2024 dataset. We also compared this framework with a conventional direct VI-based model. The trait-based model explained 89% of the variation in seed yield during calibration and showed better cross-year predictive performance than the VI-based model (R^2^ = 0.70, NRMSE = 17% versus R^2^ = 0.52, NRMSE = 22%). In addition, the model captured the decline in seed yield under excessive N input, indicating that it reflected biologically meaningful crop responses. These results demonstrated that combining structural and physiological traits can provide a more robust and interpretable alternative to conventional VI-based methods for UAV-based yield prediction. This framework has practical potential for improving precise and sustainable N management in buffalograss seed production.

## Introduction

Buffalograss (*Buchloe dactyloides* (Nutt.) Engelm.) is a warm-season perennial turfgrass widely valued for its excellent drought tolerance and low-input management requirements. These characteristics make it particularly suitable for arid and semi-arid regions, where water conservation and reduced maintenance are increasingly important (Breuillin‐Sessoms and Watkins, 2020). As a result, its cultivation has expanded in several water-limited regions. In China, this increasing adoption has raised the demand for buffalograss seed to approximately 2,000 tons (Forestry and Grassland Administration of China, 2022), making improved seed production an important practical goal. Among the agronomic factors influencing seed production, nitrogen (N) management is especially important because it directly affects plant growth, reproductive development, and final seed yield (Lemaire et al., 2008). However, defining an appropriate N fertilization strategy remains challenging, as both insufficient and excessive N input may constrain seed yield (Mena et al., 2025). In this context, accurate seed-yield prediction is valuable because it provides a quantitative basis for evaluating crop performance and optimizing N management under field conditions (Xue et al., 2024). Therefore, developing reliable methods for field-scale seed-yield prediction is essential for improving the efficiency and sustainability of buffalograss seed production.

With the advancement of remote sensing technologies, vegetation indices (VIs) have been the crucial parameter for evaluating and predicting the crop growth and yield (Féret et al., 2021), especially when using unpiloted aerial vehicle (UAV)-based (Varela et al., 2021). While researchers reported good performance of VIs for predicting crop yield (Fu et al., 2021), their use for yield prediction has severe disadvantages (Maes and Steppe, 2019). One limitation is the high multicollinearity among different indices, which often leads to redundant inputs and model instability. More importantly still, direct yield prediction based on VIs may lack physiological interpretability, thereby reducing the model’s robustness across different environments, years, and management practices. Radiative-transfer-model + crop-growth-model couplings can, in principle, link canopy spectra to biomass mechanistically, but their practical deployment in buffalograss seed systems is still prohibitive. Such frameworks demand dozens of optical and eco-physiological parameters that are largely unavailable for this species, extensive site-specific weather-soil inputs, and labor-intensive calibration, all of which amplify model uncertainty when transferred across years or locations. For buffalograss seed yield, the prediction challenge is even greater. Unlike biomass, which can often be represented more directly by canopy structure and growth vigor, buffalograss seeds are usually embedded within a low and dense canopy and are relatively sparsely distributed in space, making stable seed information difficult to obtain from conventional UAV imagery. Therefore, seed yield cannot be directly represented by canopy greenness or vegetative growth alone. Instead, its formation depends more on the coordination between structural growth and physiological changes. Accordingly, there is a need for a buffalograss seed yield prediction framework that is both physiologically interpretable and feasible for UAV-based implementation.

Seed yield is primarily shaped by the crop’s developmental and physiological status (Kemanian et al., 2007; Gemechu Asefa, 2019), such intermediate traits are more directly linked to yield formation and tend to maintain more stable relationships with yield, with relatively less interference from environmental and management variation. Therefore, estimating these yield-related intermediate traits from UAV observations may provide a more robust basis for seed-yield prediction. Recent advances in UAV remote sensing have enabled the reliable retrieval of diverse crop phenotypes for high-throughput monitoring and phenotyping. Because yield is not determined by a single dimension, these intermediate traits can be broadly classified into structural and physiological indexes, which respectively reflect crop growth architecture and nutritional–metabolic status and jointly contribute to final yield formation. At the same time, practical implementation requires consideration of sensor availability and acquisition cost. Given that conventional UAV platforms are commonly equipped with RGB and multispectral sensors, traits that are both physiologically meaningful and retrievable from these data sources are particularly attractive.

Among structural traits, plant height (PH) is a practical candidate because it can be efficiently estimated from UAV-derived digital surface model (DSM) and digital elevation model (DEM) differencing, and its retrieval from RGB-based canopy reconstruction is already well established. In buffalograss, PH provides an accessible proxy for structural growth, reflecting not only overall canopy development but also vertical architecture and biomass allocation pattern. Both excessively high and low PH may be unfavorable for seed yield formation: taller plants may allocate disproportionately more assimilates to structural tissues, increasing nutrient transport costs and reducing partitioning to reproductive organs, whereas shorter plants may indicate insufficient vegetative development and limited photosynthetic capacity. For physiological traits, nutrient-related indicators are especially relevant because seed production depends strongly on crop nutritional status and nutrient remobilization during the transition from vegetative to reproductive growth. Multispectral sensing has been widely used for nutritional diagnosis, making nutrient-related traits particularly suitable for practical UAV applications. Although total chlorophyll content, often approximated by NC × LAI, is commonly used to estimate yield potential, accurate LAI retrieval remains difficult in buffalograss because of its low, stoloniferous canopy and dense ground coverage. Under these conditions, canopy nitrogen content (CNC) is a more feasible physiological indicator, because it can be effectively characterized from multispectral information without relying on accurate LAI estimation. CNC reflects crop nutritional and physiological status, and plays a critical role in nutrient remobilization during reproductive development. However, excessive N may delay senescence by promoting prolonged vegetative growth and hormonal imbalance (Jun et al., 2023), which may in turn stimulate excessive stem elongation and further reduce assimilate distribution efficiency during seed filling (Li et al., 2023). Besides, their interaction should also be considered. Excessive nitrogen supply may promote overly vigorous vegetative growth and reduce reproductive allocation, whereas tall plants under insufficient nitrogen may lack adequate nutrient support for seed development. Similarly, excessively low plant height may indicate limited vegetative growth and source capacity. Therefore, the interaction between PH and CNC should also be considered when predicting buffalograss seed yield.

This study aimed to achieve the following objectives: (1) to analyze the responses of field-measured PH, CNC, and seed yield of buffalograss under different N application levels; (2) to establish and evaluate a trait-based PH + CNC yield model based on field measurements, and to quantify the individual and interactive contributions of PH and CNC to seed yield; and (3) to assess the scalability of the field-calibrated PH–CNC–yield model, alongside a PH + VI baseline, using independent UAV-derived data from the third-year experiment.

## Materials and methods

### Experiment area and materials

The experiment was conducted at the experimental station of China Agricultural University, located in Zhuozhou City, Hebei Province, China (39°37′N, 115°51′E) during the years 2022, 2023 and 2024. The region experiences a typical temperate continental semi-humid monsoon climate, characterized by rainfall predominantly during summer. Yearly rainfall varies between 550 and 650 mm, with 50% to 75% in July and August. The field’s root zone soil layer, extending to a depth of 40 cm, consists of sandy soil with an alkaline pH level of 8.1. Other physical and chemical characteristics of the soil are detailed in **Table 1**.

**Table 1:** Soil physical and chemical properties in the experimental site.

Soil depth (cm)	Field capacity (cm³ cm^-^³)	Soil bulk density (g cm^-^³)	Available P (mg kg^-1^)	Available K (mg kg^-1^)	Organic matter (mg kg^-1^)	Nitrate-nitrogen content (mg kg^-1^)
0-20	0.24	1.67	31.76	49.90	7.88	39.30
20-40	0.20	1.69	13.16	24.36	2.30	8.95

The buffalograss cultivar of “Zhongping No. 1” was seeded in the experimental field in May 2021 at a seeding rate of 50 kg ha^-1^ and a row spacing of 60 cm. Depending on the local management, the compound fertilizer with 45 kg N ha^−1^, 19.6 kg P ha^−1^, and 38 kg K ha^−1^ were applied as base fertilizer before sowing. During the establishment year (2021), the total rainfall was 218.2 mm, and an additional 82 mm was provided through irrigation. Soil moisture was measured with a TDR Trime-tube system (Trime-T3, IMKO, Germany) and was kept between 60% and 95% of field capacity (FC) throughout the experiment. If required, irrigation took place every third day in the morning.

### Experimental treatments

**Figure 1:**
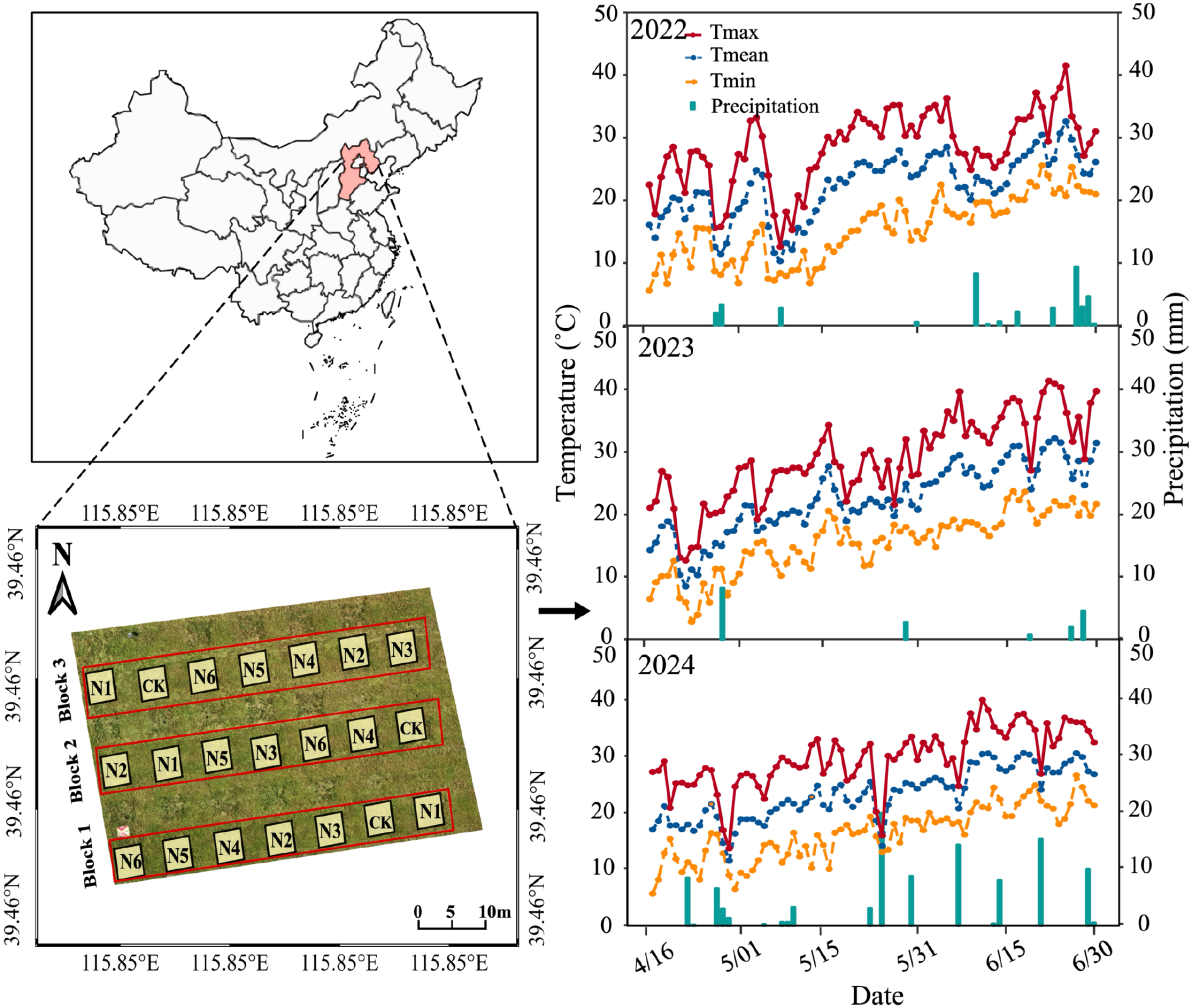
The location of the experiment plots with different nitrogen application levels and meteorological condition the region of experiment during the buffalograss growing seasons in 2022, 2023 and 2024.

**Figure 2:**
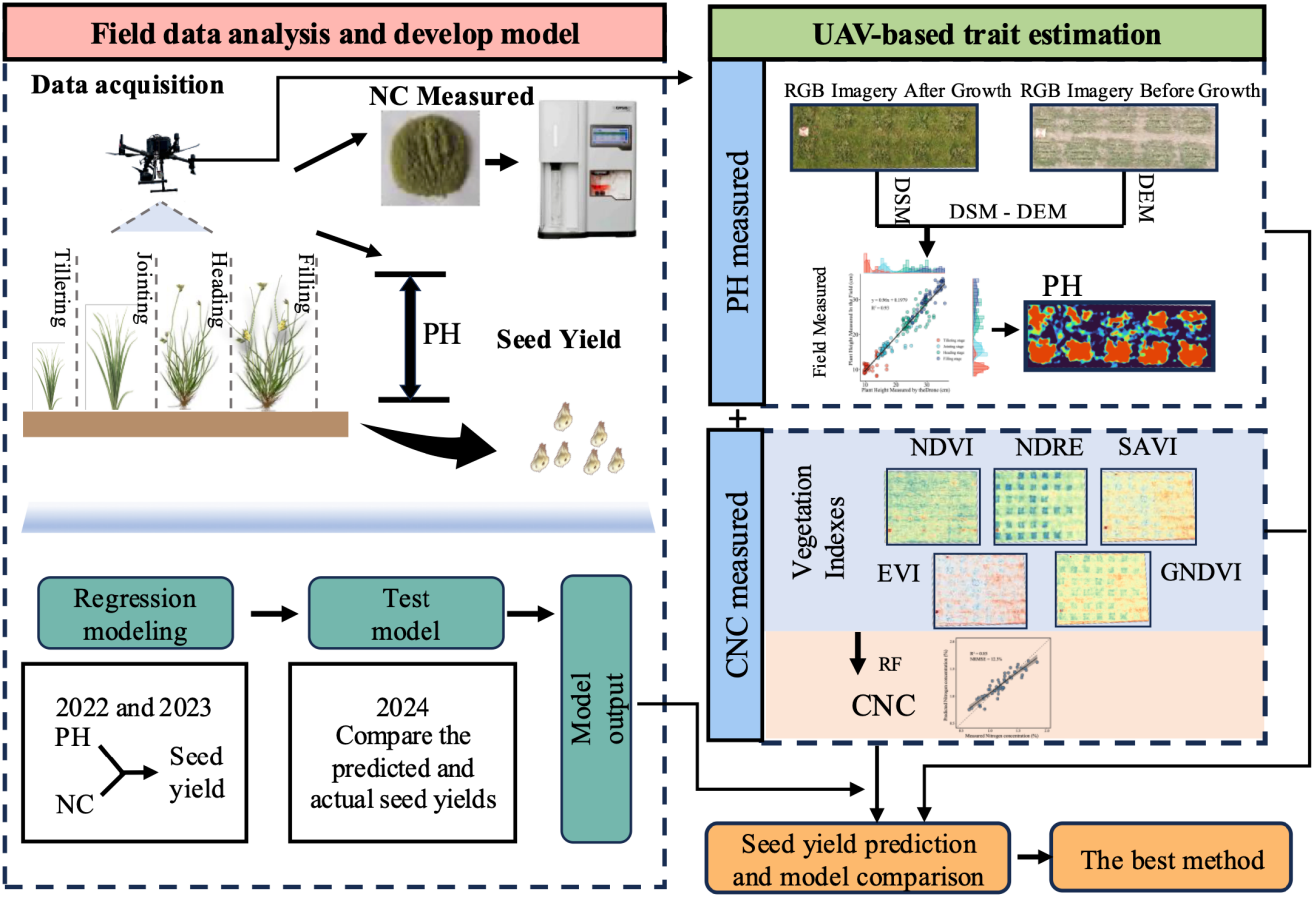
Technical flow chart of this experiment.

To align with local N fertilizer management practices and address local needs, the experiment included seven N application levels (N0–0 kg·ha^-1^, N1–60 kg·ha^-1^, N2–90 kg·ha^-1^, N3–120 kg·ha^-1^, N4–150 kg·ha^-1^, N5–180 kg·ha^-1^, N6–210 kg·ha^-1^) (**Table 2**). Considering the decrease in N demand during the reproductive growth stage, N application was applied following a 2:1 ratio, with two-thirds of the total N applied during the vegetative growth stage (tillering and jointing stages) and one-third applied during the reproductive growth stage (heading and filling stages). The field site was approximately 32 m wide and 56 m long. A randomized complete block design with three replications and a total of 21 plots was implemented approximately 4m × 4m in dimension (**Figure 1**, **Table 2**). The test flow of the experiment is shown in **Figure 2**. 

**Table 2:** The buffalograss nitrogen levels of various treatments.

Years	Stage date	N application kg·N·ha^-1^
2022	Tillering (April 25) Jointing (May 13) Heading (May 28) Filling (June 12)	0, 60, 90, 120, 150, 180, 210
2023	Tillering (April 23) Jointing (May 15) Heading (June 1) Filling (June 12)	0, 60, 90, 120, 150, 180, 210
2024	Tillering (April 20) Jointing (May 10) Heading (May 30) Filling (June 10)	0, 60, 90, 120, 150, 180, 210

### Field data

#### Meteorological data

Meteorological factors, including air temperature and precipitation were continuously monitored in the test area using a portable weather station (HOBO U30, Onset, Massachusetts, USA) installed in the study field.

#### Canopy nitrogen content

At the tillering, jointing, heading, and filling stages, buffalograss samples measuring 30 cm × 30 cm were collected from the plots. The samples were placed in an oven at 105 °C for 2 hours, and then dried at 70 °C until a constant weight was achieved. The samples were ground and then passed through a 40-mesh sieve. Eventually, the nitrogen content of samples was determined via the Kjeldahl method by the Kjeldahl apparatus (KjeltecTM 8000, Foss, Hilleroed, Denmark).

#### Plant height

At the tillering jointing, heading and filling stages, the plant height was measured directly using a ruler of five representative buffalograss plants each plot.

#### Seed yield

At harvest, the buffalograss seed was collected in a 1 × 1 m^2^ area of each plot, and sun-dried to constant weight (the water content of seed below 13%). The yield per hectare was calculated per plot.

### UAV platform and image acquisition

#### Image acquisition

A UAV (M300 RTK, DJI Technology Co., Shenzhen, China) was deployed for the aerial imagery data acquisition. RGB images were collected by the integrated camera (Zenmuse H20T, DJI Sciences and Technologies Ltd., Shenzhen, China) with three spectral brands: blue (450nm), green (520nm) and red (R, 660nm). Multispectral images were captured with a RedEdge-M multispectral camera (AgEagle, USA), with sensors in the blue (B, 465-485nm), green (G, 550-570nm), red (R, 663-673nm), red edge (RE, 712-722nm) and near infrared (NIR, 820-860nm) spectrum. Flights were performed on 8 different dates in 2022 and in 2023 and on 7 dates in 2024, corresponding with the major growing stages. A standard grey panel (Calibrated Reflectance Panel (CRP), MicaSense) was photographed for radiometric correction before each flight. All flights were executed between 10:00 am and 2:00 pm under clear sky, with no wind or clouds. The flight altitude was set to 20m above ground, and thus obtained RGB and multispectral images at ground sampling distance (GSD) of 0.7 and 3.2 cm per pixel, respectively. All flight missions were programmed using the DJI Pilot app (DJI Technology Co., Shenzhen, China), with UAV images captured at 75% overlap. Five ground control points were set up in the field as recommended (Maes, 2025) and their precise coordinates and elevation were measured using a Sinan Navigation M600 mini high-precision Global Navigation Satellite System (GNSS) receiver.

#### Image data processing

The raw images were imported into Pix4D Mapper (Pix4D Mapper, Pix4D, Inc., Lausanne, Switzerland) and georeferenced orthomosaics, DSMs and DEMs were created. The DEM was derived from a bare-ground UAV flight conducted post-mowing before the onset of the green-up stage, when vegetation height was near zero in early spring 2022. Mowing was used to accelerate the time green-up stage and effectively simulate bare-soil conditions. For every subsequent date, a new DSM was produced using identical Pix4D settings and re-projected to the DEM grid using bilinear resampling. Plant height (PH) per pixel was then computed as DSM – DEM. All ortho-mosaics were processed in QGIS V3.34 (QGIS Development Team, Open-Source Geospatial Foundation). Background (soil) pixels were masked out using the normalized difference vegetation index (NDVI) which were converted to binary images by the vegetation index threshold (OTSU) (Otsu, 1979).

### UAV-based estimation of plant height and canopy nitrogen concentration

#### Plant height

In QGIS, the PH of each acquisition was calculated by subtracting the DSM from the DEM in Equation 1. NDVI, NDRE, EVI, GDVI and SAVI were calculated in the **Table 3**.

**Table 3:** The vegetation indices derived from multispectral data used in this study.

Name	Formula	Reference
*NDVI*	*NDVI* = (*NIR* − R)/(*NIR* + *R*)	(Rouse et al., 1974)
*NDRE*	*NDRE* = (*NIR* − *RE*)/(NIR + *RE*)	(Gitelson and Merzlyak, 1997)
*EVI*	*EVI* = 2.5 × (*NIR* − *R*)/(*NIR* + 6*R* − 7.5*B* + 1)	(Huete et al., 1999)
*GNDVI*	*GNDVI* = (*NIR* − *G*)/(*NIR* + *G*)	(Gitelson et al., 1996)
*SAVI*	*SAVI* = 1.5 × (*NIR* − *R*)/(*NIR* + *R* + 0.5)	(Huete, 1988)

(1) $$\hat{P}i={\text{DSM}}_{i}-\text{DEM}$$

Where $\hat{P}i$ is the PH of i stage for each plot was calculated as the mean height of canopy pixels within the plot boundary.

### Canopy nitrogen concentration

CNC was estimated with two stage-specific RF models—one calibrated on vegetative data (tillering + jointing) and the other on reproductive data (heading + filling)—because the physiological shift after heading creates the principal change in N dynamics, whereas further subdivision into four shorts, overlapping stages would add little benefit and complicate phenological delineation. Specific models used the same VI set (NDVI, NDRE, EVI, GDVI, SAVI) and the common RF hyper-parameters (n_estimators = 50, max_depth = 6, min_samples_leaf = 3, max_features = “sqrt”, random_state = 42).

### Model development

#### Field-based seed yield modeling

To represent the joint effects of plant structural traits and nitrogen status on seed-yield formation, the yield response was decomposed into a source module, a sink module, and a coupling module in Equations 2–4. In this framework, CNC was used to characterize the source-related physiological status of the crop, based on the source–sink concept of nitrogen transport and use (Tegeder and Masclaux-Daubresse, 2018). PH was used to represent sink-related structural growth. The coupling module was formulated as a multiplicative term to describe the joint contribution of source- and sink-related traits to seed yield. This form was adopted to account for the nonlinear and coordinated effects of structural and physiological traits on yield formation, rather than assuming purely additive effects, and is consistent with the coupled nonlinear nature of crop physiological processes (Wu et al., 2016). We decomposed the yield response function into source and sink modules, and further defined their interactive contribution as follows:

Source module (CNC).

(2) $$Y_{N}=\left(\delta_{1}\text{N}+\delta_{2}{\text{N}}^{2}\right)$$

Sink module (PH).

(3) $$Y_{P}=\left(\gamma_{1}\text{P}+\gamma_{2}{\text{P}}^{2}\right)$$

Coupling module.

(4) $$\text{Y}=f\left(Y_{P},Y_{N}\right)$$

Final yield response model in Equation 5.

(5) $$\text{Y}=\left(\delta_{1}N+\delta_{2}N^{2}\right)\cdot \left(\gamma_{1}P+\gamma_{2}P^{2}\right)+\text{a}$$

where the P is the plant height coefficients δ_1_ and γ_1_capture the linear contribution of each factor; δ_2_ and γ_2_ represent the quadratic effects of nitrogen concentration and plant height, respectively, reflecting the potential diminishing returns at higher levels. The intercept a represents the baseline yield in the absence of both factors.

#### UAV-based modeling of seed yield

The seed-yield model was likewise implemented as a random-forest regressor. Input predictors comprised the plot-level plant height (PH) extracted from UAV DEM–DSM differencing and the five vegetation indices—NDVI, NDRE, EVI, GDVI and SAVI. Data from 2022 and 2023 were used as the training set, and the independent 2024 dataset was used as the validation set. For both datasets, PH and CNC were obtained separately at the tillering, jointing, heading, and filling stages. In the training set, stage-specific PH and CNC values were linked to the final seed yield of the same plot for model calibration. In the validation set, the PH and CNC values from each corresponding stage were separately input into the calibrated model to generate stage-specific seed-yield predictions. Hyper-parameter exploration reused the grid tested for CNC estimation, and the optimal setting remained identical: 50 trees (n_estimators = 50), maximum depth of six (max_depth = 6), at least three samples per terminal node (min_samples_leaf = 3), square-root feature sampling (max_features = “sqrt”), and a fixed random seed (random_state = 42).

### Data analysis and model evaluation

Statistical tests and analyses of the experimental data were carried out using Microsoft Excel (Microsoft, CA, USA), and RStudio (Inc. Boston, MA, USA) software. The seed yield for different treatments were subjected to one-way analysis of variance (ANOVA), with Levene’s test confirming homogeneity of variances (*p* > 0.05 for all variables). Treatments were separated using Fisher’s protected LSD at the 5% level.

The absolute departure of the normalized root mean square error (NRMSE) and coefficient of determination (R^2^) were used to assess the agreement between the predicted and actual seed yield and the CNC and PH of the measured vs drone-based estimate in Equations 6–10. All analyses were inferred at 5% significance.

(6) $$\begin{array}{c} R^{2}=1-\frac{\sum_{i=1}^{n}{\left(y_{i}-{\hat{y}}_{i}\right)}^{2}}{\sum_{i=1}^{n}{\left(y_{i}-\bar{y}\right)}^{2}} \end{array}$$

(8) $$\begin{array}{c} \text{NRM}S\text{E}=\frac{\sqrt{\frac{1}{n}\sum_{i=1}^{n}{\left(y_{i}-{\hat{y}}_{i}\right)}^{2}}}{\bar{y}}\times 100\% \end{array}$$

(9) $$NSE=1-\frac{\sum_{i=1}^{n}{\left(y_{i}-{\hat{y}}_{i}\right)}^{2}}{\sum_{i=1}^{n}{\left(y_{i}-\bar{y}\right)}^{2}}$$

(10) $$CCC=\frac{2r\sigma_{y}\sigma_{\hat{y}}}{\sigma_{y}^{2}+\sigma_{\hat{y}}^{2}+{\left(y_{i}-\hat{y}\right)}^{2}}$$

In which $y_{i}$ and $\hat{y_{i}}$ are the measured and predicted values at time i (i = 1, 2, …… n). $\bar{y}$ is the mean of y values, $\sigma_{y}\sigma_{\hat{y}}$ are the variance of $y_{i}$ and $\hat{y_{i}}$.

## RESULTS

### Field study on buffalograss height, canopy nitrogen content and harvest index

**Figure 3:**
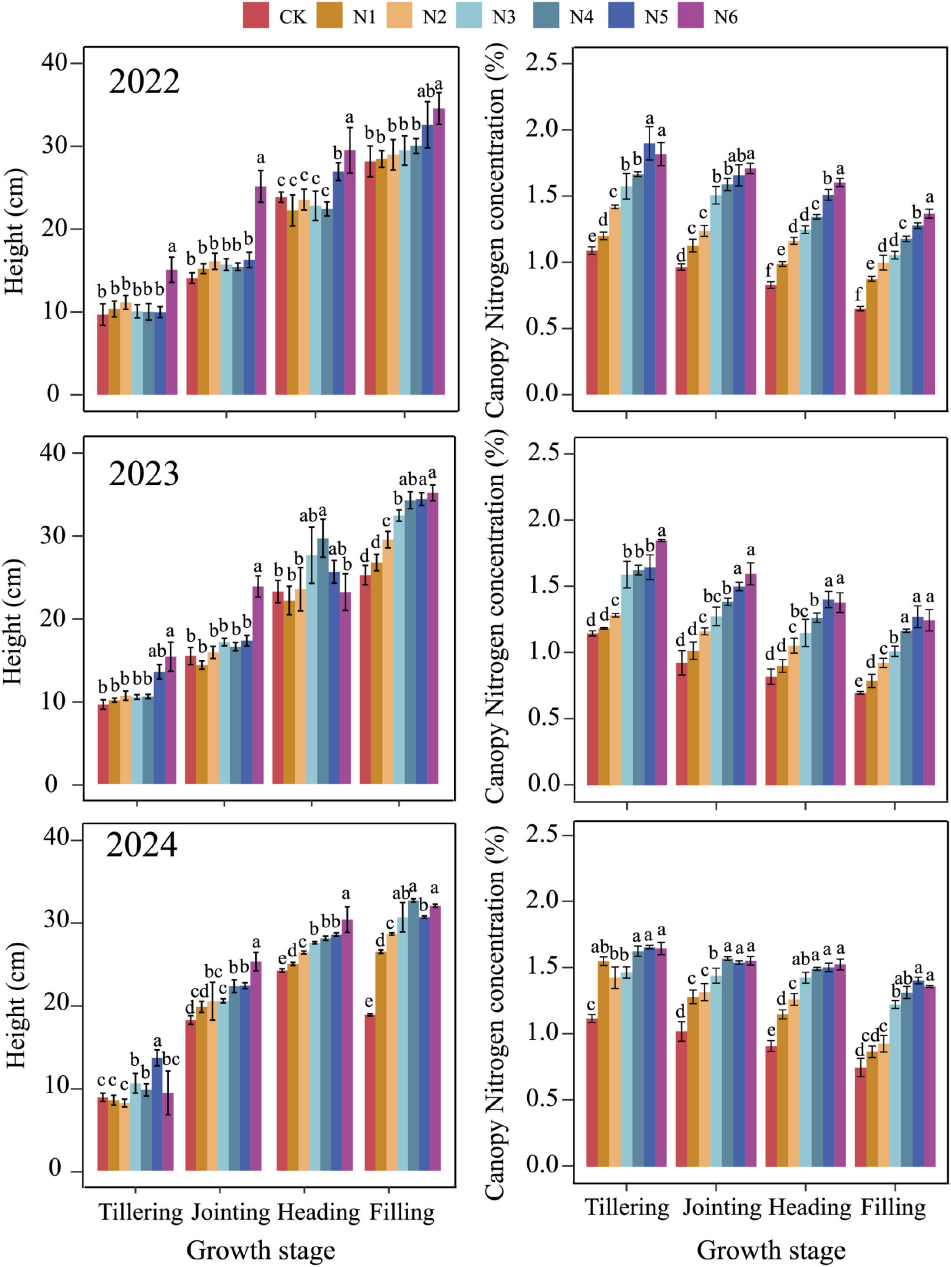
Plant height (PH) and canopy nitrogen concentration (CNC) under different nitrogen application during four growth stages in 2022, 2023 and 2024, respectively. N treatments: CK = 0 kg N ha^−1^, N1 = 30 kg N ha^−1^, N2 = 60 kg N ha^−1^, N3 = 90 kg N ha^−1^, N4 = 120 kg N ha^−1^, N5 = 150 kg N ha^−1^, N6 = 180 kg N ha^−1^. Different letters above bars indicate significant differences among treatments (*p* < 0.05).

**Figure 3** depicts the measured buffalograss PH and CNC under the different N treatments for the four growth stages over the three experimental years. The PH increased with the growth stage, with the strongest increment between the jointing and heading stages. Statistically significant differences were observed between the different N treatments, with the highest level of fertilization (N6) having the tallest plants. Differences typically increased in the course of the growing season, with up to five significantly different levels in 2024.

The effect of nitrogen treatment on CNC was similar, but the seasonal pattern was clearly different, with CNC reaching its highest level at the tillering stage, followed by a slow decrease. Strong statistical differences between the treatments were already apparent from the tillering stage, and remained or intensified, with up to 6 statistically different levels in the filling stage in 2022.

### Seed yield in relation to nitrogen rate, plant height, and nitrogen content

**Figure 4:**
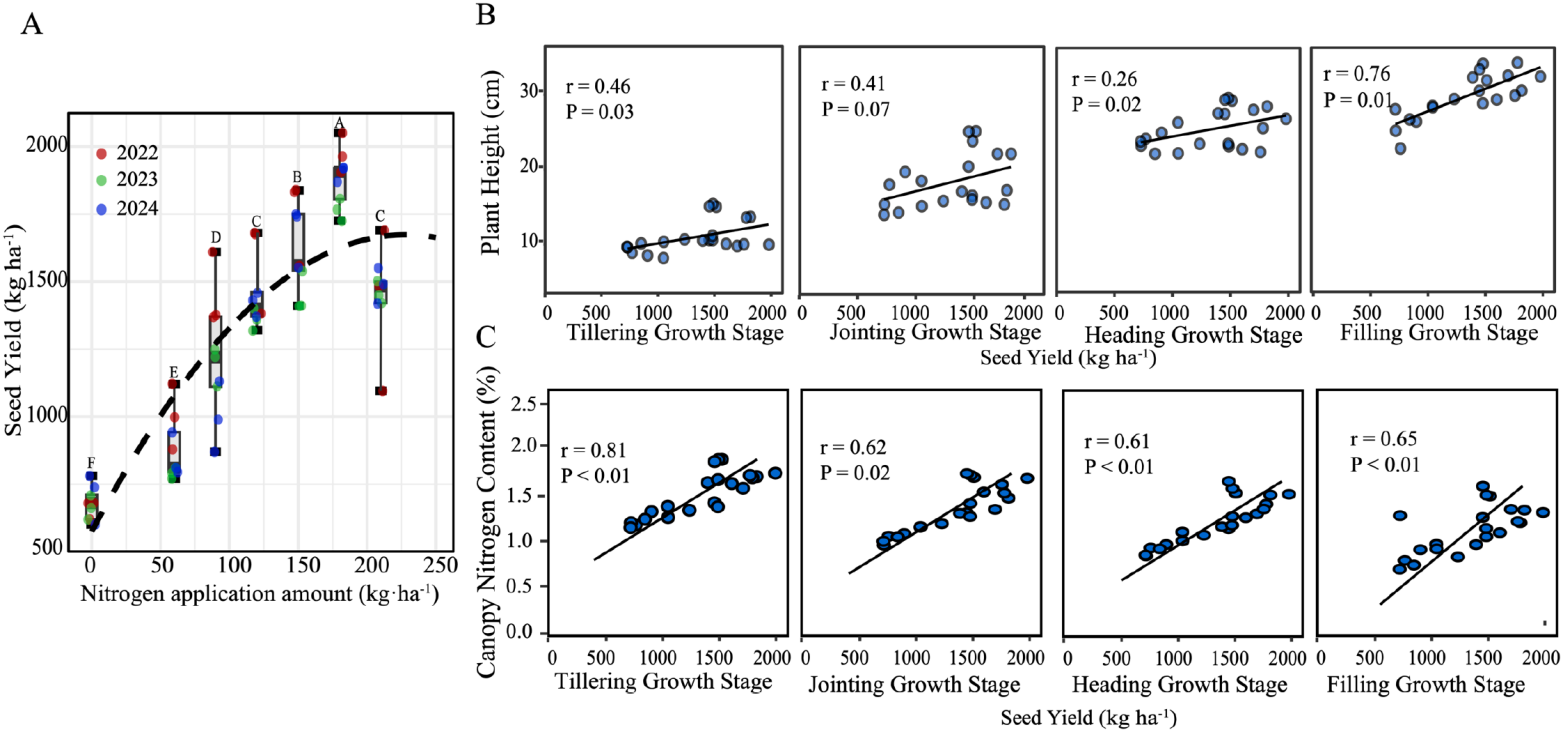
**(A)** The effect of nitrogen (N) application on seed yield across three experimental years. **(B)** Relationship between plant height (PH), canopy nitrogen content (CNC) and the seed yield for the different growing stages.

**Figure 4** illustrates the effect of N application and plant traits on seed yield across the three experimental years. Seed yield exhibits a significant nonlinear response to increasing N rates (**Figure 4**), with a distinct hump-shaped pattern. The highest yield is observed at an application rate of 180 kg·ha^−1^, beyond which further N addition led to a yield decline. This indicates an optimal N threshold for maximizing reproductive output in buffalograss. Both PH and CNC show positive linear correlations with seed yield at most stages, with CNC generally exhibiting stronger correlations compared to PH, except in the filling stage. Moreover, plant height and nitrogen content exhibited opposite trends over time—plant height generally increases with growth, while nitrogen content declines. This suggests that these two traits reflect complementary aspects of plant development, and their joint variation may contribute to explaining yield variation across stages. Therefore, we further evaluated the predictive performance of combined PH and CNC metrics in both field-measured and UAV-estimated datasets.

### Yield model based on plant height and canopy nitrogen content and validation across years

**Figure 5:**
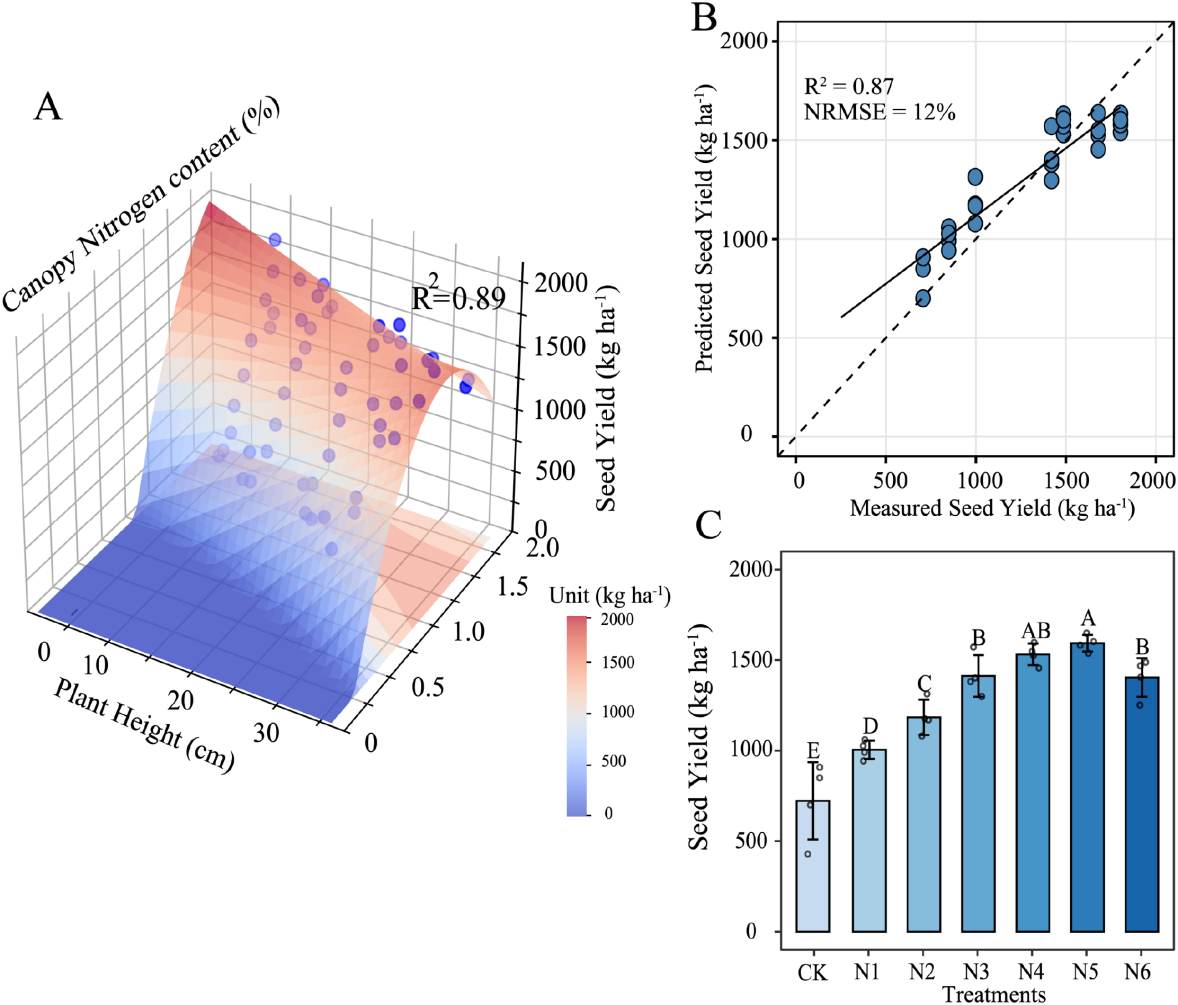
**(A)** Modelled relation between seed yield by plant height (PH) and canopy nitrogen concentration (CNC). based on 2022 and 2023 data. **(B)** Relationship between observed and predicted seed yield based on 2024 data. **(C)** Predicted seed yield for the six N-rate treatments (CK = 0 kg N ha^−1^, N1 = 30 kg N ha^−1^, N2 = 60 kg N ha^−1^, N3 = 90 kg N ha^−1^, N4 = 120 kg N ha^−1^, N5 = 150 kg N ha^−1^, N6 = 180 kg N ha^−1^.) in 2024; bars sharing a letter do not differ significantly (*p* < 0.05).

**Figure 6:**
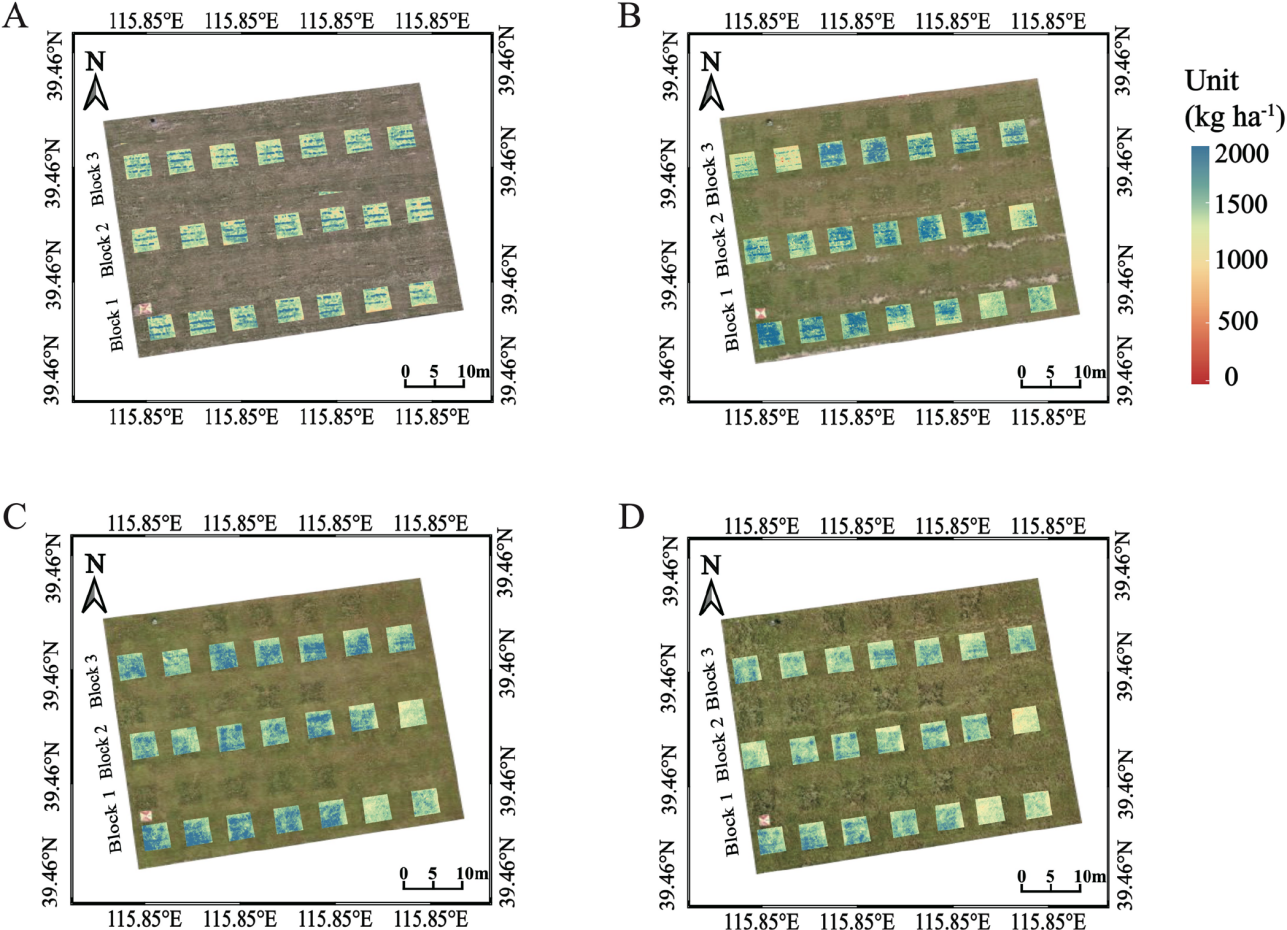
Seed yield prediction based on UAV-estimated PH and CNC averaged across all growth stages. **(A)** tillering stage; **(B)** jointing stage; **(C)** heading stage; **(D)** filling stage.

The binary quadratic function was established using PH and CNC as input variables showed as three-dimensional figure (**Figure 5A**). The result of fitting effect was relatively satisfactory, with R^2^ of 0.89. Yield increased with CNC up to an intermediate optimum 0.8 - 1.2%, after which further N depressed yield; same trend was observed for PH, with yield peaking at 10–25 cm then declining. The contribution analysis showed that CNC explained 59.5% of the yield variability, while PH contributed 13.5% (**Table 4**), indicating that CNC had a greater direct influence on seed yield. The interaction between PH and CNC accounted for 27.0% of the variability and exerted a negative effect, suggesting that increases in both variables simultaneously weakened seed yield potential. Applying the model to the third year of field data, the test results in **Figure 5B** performed well with R^2^ = 0.87 and NMRSE = 12%. Model predictions for the six N-rate treatments (**Figure 5C**) increased from CK to N3, plateaued at N3–N4, and declined significantly at the highest rate N6, correctly reflecting the yield penalty of over-fertilization. Spatial prediction maps based on UAV-estimated PH and CNC further showed clear plot-level yield variability across growth stages (**Figure 6**). This proved that modeling developed by PH and CNC was stable across years.

**Table 4:** Coefficients and contribution of PH, NC, and their interaction to the yield prediction model.

Variable		Coefficient	Contribution rate
PH	PH	135.90	13.5%
	PH^2^	-0.15	
CNC	CNC	7269.50	59.5%
	CNC^2^	-1710.00	
PH×CNC	–	-83.80	27.0%

### Comparison of seed yield predictions from different methods

**Figure 7:**
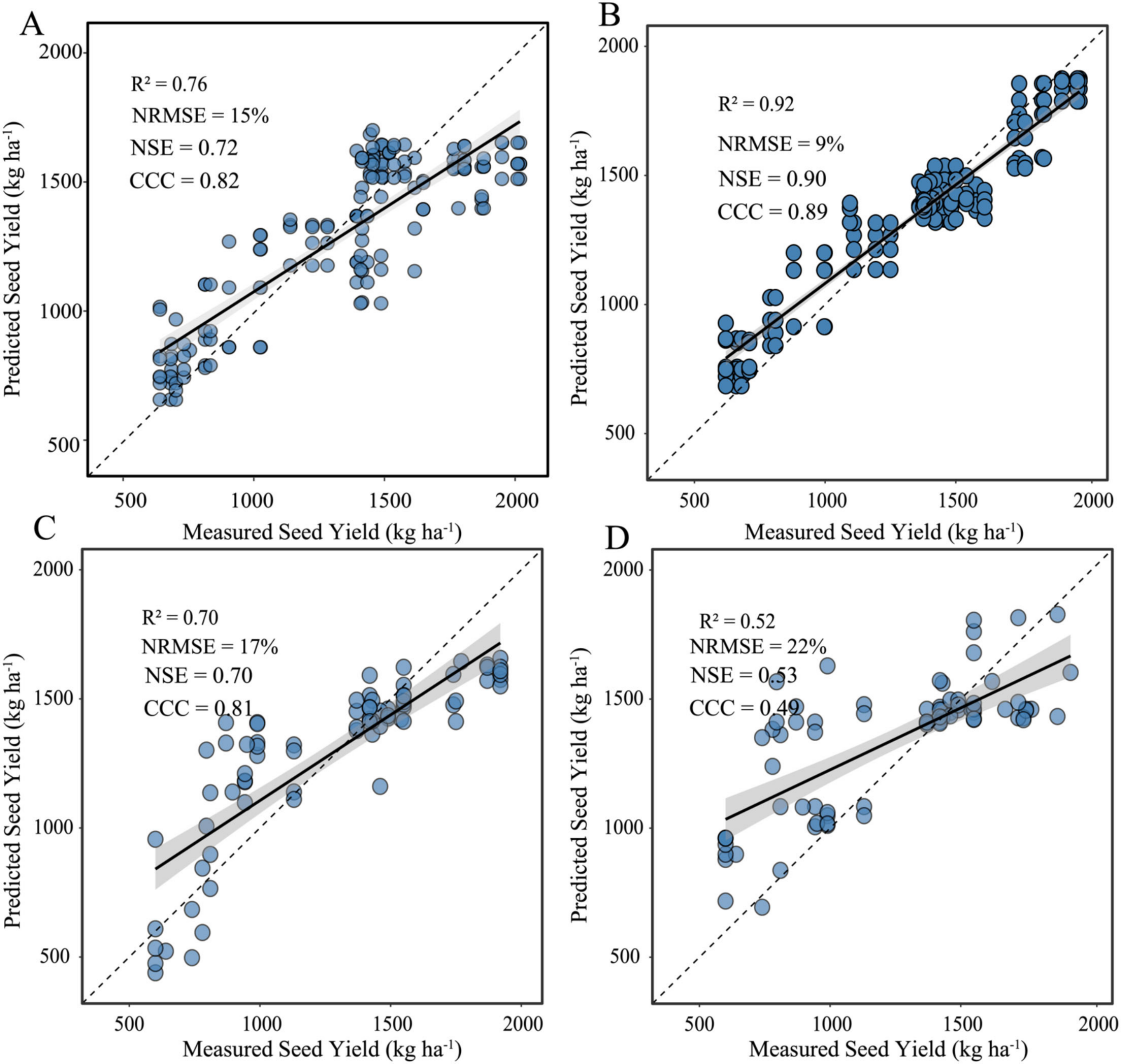
**(A)** Trait-based field model (UAV-derived PH + CNC) fitted with 2022–2023 data. **(B)** Direct yield model from UAV vegetation indices and PH fitted with the same 2022–2023 data. **(C)** Independent-year test of the field model using the 2024 data set. **(D)** Independent-year test of the spectral UAV model using the 2024 data set.

Using the 2022–2023 training data, we compared the performance of the trait-based and direct prediction models. The direct VIs + PH model (B) attains the higher in-sample accuracy (R^2^ = 0.92, NRMSE = 9%), whereas the trait model based on PH + CNC (A) peaks at R^2^ = 0.76 and NRMSE = 15%. However, when evaluated on the independent 2024 test data, the trait model showed more stable performance with R^2^ = 0.70 and NRMSE = 17%. While the direct approach loses precision with higher NRMSE = 22% and lower R^2^ = 0.52. Thus, although the direct model fits the calibration years slightly better, the PH + CNC framework offers the more stable performance across years. Furthermore, spatial prediction maps (**Figure 7**) based on UAV-estimated PH and CNC highlight the model’s capacity to visualize yield variability across the field. Seed yield differences among plots were clearly distinguishable, reflecting the model’s robustness in translating physiological traits into spatially explicit yield predictions.

## Discussion

In this study, PH and CNC emerged as two key traits associated with seed yield formation across growth stages under varying nitrogen treatments. PH exhibited a delayed response to nitrogen availability (**Figure 3**), reflecting cumulative nutrient assimilation over the growth cycle (Ciampitti and Vyn, 2012). Unlike CNC, which responded rapidly to changes in nitrogen application rates (Yue et al., 2021), PH increased more gradually and stabilized after the early vegetative stages. Meanwhile, CNC reached a saturation threshold under excessive nitrogen supply (**Figure 3**), beyond which additional nitrogen no longer promoted seed yield and potentially caused yield reduction. In contrast, PH maintained clear differentiation among treatments, providing consistent structural information complementary to the physiological signals from CNC. Individually, both PH and CNC showed positive correlations with yield (**Figure 4**), but their combination significantly enhanced the predictive capacity of the model. This improvement arises because PH and CNC characterize distinct yet interconnected physiological processes—structural biomass accumulation versus nutrient assimilation and allocation—thus offering a more holistic representation of yield-determining factors. The differences in seed yield among years in **Figure 4A** were mainly related to variation in meteorological conditions. Because irrigation was applied according to percentages of field capacity, the effect of precipitation was relatively small. Temperature was therefore the main climatic factor considered here. Seed yield was highest in 2022. The mean growing-season temperature in 2022 was 22.3 °C, which was not the highest among the three years. However, this moderate temperature was more favorable for the coordination between vegetative and reproductive growth. No abnormal high-temperature events occurred before or after heading. In addition, the relatively warm conditions during the vegetative stage may have promoted earlier reproductive development. As a result, heading occurred earliest in 2022 (May 28). This allowed flowering, seed set, and early grain filling to partially avoid the higher temperatures later in the season, which favored seed formation. In 2023, the mean temperature was the lowest (21.9 °C), and heading was latest (June 1). This suggests insufficient heat accumulation during the early growth stage. At the same time, the reproductive stage was more likely to be exposed to high temperatures. This was unfavorable for seed set and grain filling. Therefore, seed yield was lowest in 2023.In 2024, the mean temperature was the highest (23.0 °C). Earlier tillering and jointing indicated better thermal conditions during the vegetative stage. However, the relatively high hydrothermal conditions may have promoted excessive vegetative growth and accelerated crop development. The reproductive stage was also exposed to stronger heat conditions, with more hot days. As a result, seed yield in 2024 was higher than that in 2023, but lower than that in 2022.

The complementary physiological roles of PH and CNC underpin their strong joint contribution to yield prediction. NC represents the source of assimilates, closely tied to photosynthetic capacity, while PH symbolizes the assimilate sink, indicating structural investment and potential grain-bearing sites (Lin et al., 2020). When CNC reaches photosynthetic saturation, additional nitrogen accumulates in vegetative tissues, becoming unproductive or even detrimental to reproductive development (Cossani et al., 2012). Similarly, excessive plant height (beyond approximately 25–30 cm) raises respiratory costs and lodging risks, outweighing its yield-enhancing benefits. Consequently, the quadratic yield-response surface incorporating both PH and CNC (**Figure 5A**) effectively captured this source–sink dynamic, explaining 89% of the yield variability in 2022–2023 and retaining robust predictive accuracy (R^2^ = 0.87) in the independent 2024 dataset. Importantly, the model successfully identified the yield decline resulting from nitrogen oversupply (N6, **Figure 5C**), indicating its ability to capture adverse physiological responses to excessive fertilization. The negative PH × CNC interaction term pinpointed the physiological condition wherein high nitrogen content coincides with excessive vegetative growth, causing carbohydrate diversion toward structural tissues rather than seeds (Anas et al., 2020). In contrast, maximum yields corresponded to moderate nitrogen availability paired with adequate structural growth, highlighting the necessity of maintaining a balanced source–sink relationship rather than extremes of either trait alone.

The integration of PH and CNC into the yield estimation model provided distinct advantages compared to traditional spectral indices. As structural and physiological traits, PH and CNC were biologically meaningful, directly reflecting seed yield formation processes (Burgess et al., 2023; Herr et al., 2023). The direct UAV-based prediction model performed well on training datasets (R^2^ = 0.92, NRMSE = 9%; **Figure 7B**), mainly due to its flexibility and larger set of spectral variables, which allowed it to capture year-specific variability. However, this flexibility also made it more susceptible to variations in environmental conditions, such as light, soil background, and crop phenology, thus causing accuracy to decline significantly when predicting in independent years (R^2^ = 0.52, NRMSE = 22%; **Figure 7D**). In contrast, the PH + CNC model employed a more constrained and biologically interpretable structure, enhancing stability across growing seasons. Statistically, the model’s two-step design replaced multiple correlated spectral indices with the single, robust physiological trait CNC, thus minimizing spectral noise. Additionally, the fixed quadratic formulation reduced the risk of overfitting. These physiological boundaries embedded within the model allowed it to reliably capture the adverse impact of excessive nitrogen applications (evidenced by yield reductions in high-N treatments like N6) and maintain consistent predictive accuracy across multiple seasons in **Figure 7C** (R^2^ = 0.70, NRMSE = 17%). Thus, despite slightly lower in-sample accuracy compared to the direct spectral approach, the PH + CNC model delivered superior cross-year stability, indicating its practical applicability in precision agricultural management.

This study, however, has certain limitations. Our CNC estimates depend heavily on chlorophyll-based VIs. Chlorophyll content naturally declines during reproductive stages, particularly during grain filling, even though total plant nitrogen may remain relatively high (Zhang et al., 2021; Wang et al., 2023). Consequently, this chlorophyll decline could result in underestimation of seed yield during late-season predictions. Employing advanced, high-resolution sensing technologies, such as hyperspectral imaging or LiDAR, could mitigate this issue by providing more detailed physiological and structural data. Nevertheless, the current high cost and operational complexity of these advanced sensors limit their widespread adoption in routine agricultural practices (Chen et al., 2025). Additionally, the models developed and validated in this study were confined to a single experimental site. To ensure generalizability and robustness across diverse agricultural settings, future research should incorporate data collected from multiple locations and a wider range of environmental conditions.

## Conclusions

In this study, we established a physiologically interpretable seed yield prediction model for buffalograss using UAV-derived PH and CNC based on three years of field experiments. The results demonstrated that PH and NC represent complementary aspects of yield formation, functioning respectively as indicators of structural biomass and nutrient assimilation. The developed PH + CNC model effectively captured their physiological interplay, explaining 89% of yield variability. Compared to traditional approaches directly utilizing multiple vegetation indices, the trait-based PH + CNC model provided greater robustness, especially during independent validation with R^2^ = 0.70, NRMSE = 17%. This study emphasizes the importance of integrating interpretable physiological traits into yield prediction frameworks and provides a reliable and stable method for UAV-based remote sensing techniques accurate to predict seed yield in buffalograss across years.

## Data Availability

The raw data supporting the conclusions of this article will be made available by the authors, without undue reservation.
